# Supplementary material for: Safety, tolerability, and clinical outcomes of hydroxychloroquine for hospitalized patients with coronavirus 2019 disease
Source: PLoS One. 2020 Jul 23;15(7):e0236778. doi: 10.1371/journal.pone.0236778 (PMC7377460; doi:10.1371/journal.pone.0236778)
Supplement: S1 Table — (DOCX) [file pone.0236778.s001.docx]

**S1 Table. Classification of hypoxia according to Sequential Organ Failure Assessment (SOFA) score criteria^1^**

| **Hypoxia Score** | **PaO_2_/FIO_2_** | **SpO_2_/FIO_2_** |
| --- | --- | --- |
| Discharge | N/A | N/A |
| 0 | >400 | >400 |
| 1 | 300-400 | 315-400 |
| 2 | 200-300 | 235-315 |
| 3^2^ | 100-200 | 135-235 |
| 4^2^ | <100 | <135 |
| Death | N/A | N/A |

Abbreviations: FIO_2_, fraction of inspired oxygen; N/A, not applicable; PaO_2_, partial pressure of oxygen (mm Hg); SpO_2_, peripheral capillary oxygen saturation.

^1^PaO_2_/FIO_2_ was used to establish the hypoxia score if arterial blood gas results were available. Otherwise, the SpO_2_/FIO_2_ ratio was used.

^2^In order to be considered for scores 3-4, the patient must have been receiving mechanical ventilation.
