# Supplementary material for: Safety, tolerability, and clinical outcomes of hydroxychloroquine for hospitalized patients with coronavirus 2019 disease
Source: PLoS One. 2020 Jul 23;15(7):e0236778. doi: 10.1371/journal.pone.0236778 (PMC7377460; doi:10.1371/journal.pone.0236778)
Supplement: S2 Table — (DOCX) [file pone.0236778.s002.docx]

**S2 Table. Conversions of supplemental oxygen into FIO_2_ (fraction of inspired oxygen)**

| **Supplemental oxygen** | **FIO_2_** |
| --- | --- |
| Room air | 21% |
| 1 liter NC | 23% |
| 2 liters NC | 25% |
| 3 liters NC | 27% |
| 4 liters NC | 30% |
| 5 liters NC | 35% |
| 6-7 liters NC | 40% |
| 8-10 liters NC | 49% |
| Non-rebreather mask | 65% |
| High-flow oxygen supplementation, CPAP, or BiPAP | As indicated on device |

Abbreviations; BiPAP, bilevel positive airway pressure; CPAP, continuous positive airway pressure; NC, nasal cannula.
