# Supplementary material for: Safety, tolerability, and clinical outcomes of hydroxychloroquine for hospitalized patients with coronavirus 2019 disease
Source: PLoS One. 2020 Jul 23;15(7):e0236778. doi: 10.1371/journal.pone.0236778 (PMC7377460; doi:10.1371/journal.pone.0236778)
Supplement: S3 Table — (DOCX) [file pone.0236778.s003.docx]

**S3 Table. Baseline factors associated with improvement in SOFA hypoxia score during the 10 days after treatment with HCQ.**

| **Variable** | **Improvement in SOFA hypoxia score (n=73)** | **Stable or worsening of SOFA hypoxia score (n=68)** | ***P*** |
| --- | --- | --- | --- |
| **Age, years** | **57 (40-68)** | **67 (55-77)** | **0.0006** |
| **Age ≥65 years** | **23 (32)** | **40 (60)** | **0.001** |
| BMI | 28.0 (24.5-34.3) | 27.5 (24.2-32.3) | 0.34 |
| Obesity (BMI >30) | 32 (44) | 24 (35) | 0.30 |
| Female gender | 30 (41) | 22 (33) | 0.28 |
| Race |  |  |  |
| White | 24 (33) | 20 (29) | 0.66 |
| Black | 8 (11) | 3 (4) | 0.15 |
| Asian | 11 (15) | 15 (22) | 0.29 |
| Hispanic ethnicity | 22 (30) | 19 (28) | 0.77 |
| Comorbidities |  |  |  |
| Coronary artery disease | 14 (19) | 13 (19) | 0.99 |
| Congestive heart failure | 7 (10) | 4 (6) | 0.41 |
| Cerebrovascular disease | 3 (4) | 7 (10) | 0.20 |
| Diabetes | 22 (30) | 14 (21) | 0.19 |
| Hypertension | 36 (49) | 35 (51) | 0.80 |
| Chronic pulmonary disease | 25 (34) | 20 (29) | 0.54 |
| COPD | 4 (5) | 7 (10) | 0.29 |
| Asthma | 14 (19) | 7 (10) | 0.14 |
| Chronic kidney disease | 8 (11) | 8 (12) | 0.88 |
| Cancer | 3 (4) | 5 (7) | 0.41 |
| Transplant | 5 (7) | 3 (4) | 0.53 |
| Current smoker | 1 (1) | 4 (6) | 0.20 |
| Former smoker | 18 (25) | 16 (24) | 0.88 |
| Healthcare worker | 5 (7) | 2 (3) | 0.44 |
| Residence at home | 68 (93) | 58 (85) | 0.13 |
| ACE/ARB use as an outpatient | 17 (23) | 16 (24) | 0.97 |
| **NSAID use as an outpatient** | **12 (16)** | **22 (32)** | **0.027** |
| Statin use as an outpatient | 23 (32) | 29 (43) | 0.17 |
| Immunosuppression medicines as an outpatient | 7 (10) | 14 (21) | 0.067 |
| Prednisone as an outpatient | 4 (5) | 10 (15) | 0.07 |
| Tacrolimus as an outpatient | 4 (5) | 1 (1) | 0.20 |
| Mycophenolate as an outpatient | 3 (4) | 1 (1) | 0.62 |
| Fever as presenting symptom | 54 (74) | 51 (75) | 0.89 |
| Cough as presenting symptom | 63 (86) | 55 (81) | 0.38 |
| Dyspnea as presenting symptom | 51 (70) | 40 (59) | 0.17 |
| Nausea or vomiting as presenting symptom | 14 (19) | 12 (18) | 0.82 |
| Diarrhea as presenting symptom | 19 (26) | 15 (22) | 0.58 |
| Duration of symptoms, days | 5 (3-8) | 7 (3-10) | 0.30 |
| Chest x-ray findings |  |  |  |
| Clear | 10 (14) | 6 (9) | 0.36 |
| Unilateral infiltrates | 11 (15) | 17 (25) | 0.14 |
| Bilateral infiltrates | 45 (62) | 41 (60) | 0.87 |
| Co-infection with other respiratory virus | 2 (3) | 2 (3) | 1.00 |
| Statin as an inpatient | 18 (25) | 17 (25) | 0.96 |
| ACEI/ARB as inpatient | 3 (4) | 6 (9) | 0.25 |
| NSAID as inpatient | 17 (23) | 17 (25) | 0.81 |
| Oseltamivir therapy | 2 (3) | 2 (3) | 1.00 |
| Azithromycin therapy^1^ | 11 (15) | 15 (22) | 0.29 |
| Ceftriaxone therapy^1^ | 21 (29) | 16 (24) | 0.48 |
| Doxycycline therapy^1^ | 11 (15) | 10 (15) | 0.95 |
| Days until initiation of HCQ | 1 (1-2) | 1 (1-2) | 0.84 |
| Fever upon start of HCQ | 36 (49) | 42 (62) | 0.14 |
| Heart rate upon start of HCQ | 96 (83-107) | 98 (84-106) | 0.68 |
| **Respiratory rate upon start of HCQ** | **20 (18-26)** | **24 (20-30)** | **0.003** |
| **Tachypnea (respiratory rate ≥22 breaths per min)** | **31 (43)** | **43 (63)** | **0.014** |
| **Lowest systolic blood pressure (SBP) upon start of HCQ, mm Hg** | **109 (98-117)** | **99 (86-115)** | **0.003** |
| **Hypotension (SBP ≤100 mm Hg)** | **23 (31)** | **38 (56)** | **0.004** |
| **Invasive mechanical ventilation** | **20 (27)** | **32 (47)** | **0.016** |
| Non-invasive oxygen supplementation | 36 (49) | 28 (41) | 0.33 |
| White blood cell count at HCQ initiation | 5.9 (4.3-7.6) | 7 (4.5-9.6) | 0.08 |
| **Absolute lymphocyte count (ALC) at HCQ initiation, in 10^9^ cells/µL** | **0.9 (0.6-1.2)** | **0.8 (0.5-1.1)** | **0.02** |
| **Lymphopenia (ALC <1x10^9^ cells/µL)** | **38 (52)** | **47 (69)** | **0.039** |
| Hemoglobin at HCQ initiation, g/dL | 13 (12.2-14.3) | 12.8 (11.1-13.8) | 0.11 |
| Platelet count at HCQ initiation, 10^9^/L | 192 (151-226) | 174 (127-216) | 0.11 |

Variables are expressed as No. (%) or median (IQR), unless otherwise indicated.

Abbreviations: ACE, angiotensin-converting enzyme inhibitor; ARB, angiotensin II receptor blocker; BMI, body mass index; NSAID, non-steroidal anti-inflammatory drug; SOFA, Sequential Organ Failure Assessment;

^1^Included patients who received the antibacterial agent for ≥3 days and at the same time as HCQ initiation.
